# Dendritic Cells Transduced to Express Interleukin 4 Reduce Diabetes Onset in Both Normoglycemic and Prediabetic Nonobese Diabetic Mice

**DOI:** 10.1371/journal.pone.0011848

**Published:** 2010-07-29

**Authors:** Melanie A. Ruffner, Paul D. Robbins

**Affiliations:** 1 Department of Microbiology and Molecular Genetics, University of Pittsburgh School of Medicine, Pittsburgh, Pennsylvania, United States of America; 2 Department of Bioengineering, University of Pittsburgh School of Medicine, Pittsburgh, Pennsylvania, United States of America; University of Bremen, Germany

## Abstract

**Background:**

We and others have previously demonstrated that treatment with bone marrow derived DC genetically modified to express IL-4 reduce disease pathology in mouse models of collagen-induced arthritis and delayed-type hypersensitivity. Moreover, treatment of normoglycemic NOD mice with bone marrow derived DC, genetically modified to express interleukin 4 (IL-4), reduces the onset of hyperglycemia in a significant number of animals. However, the mechanism(s) through which DC expressing IL-4 function to prevent autoimmune diabetes and whether this treatment can reverse disease in pre-diabetic NOD mice are unknown.

**Methodology/Principal Findings:**

DC were generated from the bone marrow of NOD mice and transduced with adenoviral vectors encoding soluble murine IL-4 (DC/sIL-4), a membrane-bound IL-4 construct, or empty vector control. Female NOD mice were segregated into normoglycemic (<150mg/dL) and prediabetic groups (between 150 and 250 mg/dL) on the basis of blood glucose measurements, and randomized for adoptive transfer of 10^6^ DC via a single i.v. injection. A single injection of DC/sIL-4, when administered to normoglycemic 12-week old NOD mice, significantly reduced the number of mice that developed diabetes. Furthermore, DC/sIL-4, but not control DC, decreased the number of mice progressing to diabetes when given to prediabetic NOD mice 12–16 weeks of age. DC/sIL-4 treatment also significantly reduced islet mononuclear infiltration and increased the expression of FoxP3 in the pancreatic lymph nodes of a subset of treated animals. Furthermore, DC/sIL-4 treatment altered the antigen-specific Th2:Th1 cytokine profiles as determined by ELISPOT of splenocytes in treated animals.

**Conclusions:**

Adoptive transfer of DC transduced to express IL-4 into both normoglycemic and prediabetic NOD mice is an effective treatment for T1D.

## Introduction

Type I diabetes (T1D) is a polygenic autoimmune disease characterized by destruction of insulin-secreting β-cells in the islets of Langerhans by a chronic inflammatory infiltrate. In both humans and the nonobese diabetic (NOD) mouse, destructive insulitis is predominantly a T-cell mediated phenomenon, but other cells types, such as NKT cells and antigen presenting cells (APC), also play important roles in modulating disease onset and progression [1], [2], [3]. A combination of factors including a Th1-skewed CD4+ response as well as a deficiency of regulatory T cells are considered to be important hallmarks of disease progression [1].

A shift in the Th1/Th2 balance of T cell responses towards characteristic Th1 properties, such as the release of IFN-γ, plays an important role in the initiation and progression of the inflammatory response in T1D. Conversely, shifting the balance of CD4+ responses towards a Th2-mediated response has a therapeutic effect. Delivery of exogenous IL-4, which polarizes T cell responses towards a Th2 phenotype, has been demonstrated to be an effective treatment of T1D in murine models. Systemic administration of recombinant IL-4 in NOD mice increases Th2-mediated responses in the spleen, thymus and pancreatic islets [4]. Transgenic NOD mice expressing IL-4 in the pancreatic islets are protected from the development of diabetes. Similarly, we have reduced onset of hyperglycemia in NOD mice after regulated delivery of IL-4 to pancreatic β cells *in vivo* using an adenoassociated vector (AAV) expressing IL-4 under the control of the mouse insulin promoter [5]. Furthermore, we and others have demonstrated that adoptive transfer of dendritic cell (DC) transduced *ex vivo* with IL-4-expressing adenoviral and retroviral vectors traffic to the spleen and pancreatic lymph node of NOD mice and are capable of preventing diabetes onset [6], [7].

DC are APC which play a critical role in the development of central tolerance and the maintenance of peripheral tolerance by presenting foreign- or self-derived antigens in the context of instructive secondary signals to T cells. Results from both NOD mice and patients with type 1 diabetes document abnormalities in DC function such as increased NF-κB activity, decreased expression of indoleamine-2,3-dioxygenase, and altered costimulatory and cytokine secretion profiles [8], [9], [10], [11]. Further, DC are responsible for the presentation of islet-cell derived antigens to diabetogenic T cells as well as to regulatory T cell populations within the pancreas and pancreatic lymph node [12], [13], [14]. In addition to genetic modification to express exogenous IL-4, the therapeutic benefit of other DC modifications, including expression of galectin-1, treatment with NF-κB-specific oligodeoxyribonucleotide, and antisense oligonucleotide-mediated blockade of costimulatory molecule expression has been demonstrated [15], [16], [17]. A Phase I study testing DC treated with anti-sense oligonucleotides to reduce expression of co-stimulatory molecules in type 1 diabetes patients is underway.

In the NOD mouse model, insulitis typically begins around 3–4 weeks of age, and diabetes occurs in 60–80% of female mice between 12–18 weeks of age depending on the housing conditions. The NOD model has been used to test a large number of potential therapeutic interventions, many of which have ultimately had little to no clinical benefit [18]. Although many interventions are successful in preventing diabetes onset in younger mice, they fail to prevent diabetes in older NOD mice with more advanced insulitis. Therefore, in our studies we were interested in determining the therapeutic potential of DC in later stages of disease progression in the NOD mouse.

Here we demonstrate that DC/sIL-4 treatment delays or prevents diabetes onset in a significant number of 12 week old normoglycemic mice, induces changes in the balance of Th2 vs Th1 effector profiles in splenocytes of treated mice, and induces upregulation of FoxP3 expression in the PLN of a subset of mice. We further demonstrate that DC/sIL-4 reduce the progression to overt hyperglycemia when administered to 12–16 week-old prediabetic NOD mice that already have impaired responses to glucose challenge. Taken together, our results indicate that DC/sIL-4 ameliorates islet destruction in late stages of insulitis through modulation of T cell responses.

## Results

### Characterization of DC transduced with IL-4-expressing vectors

**Figure 1:**
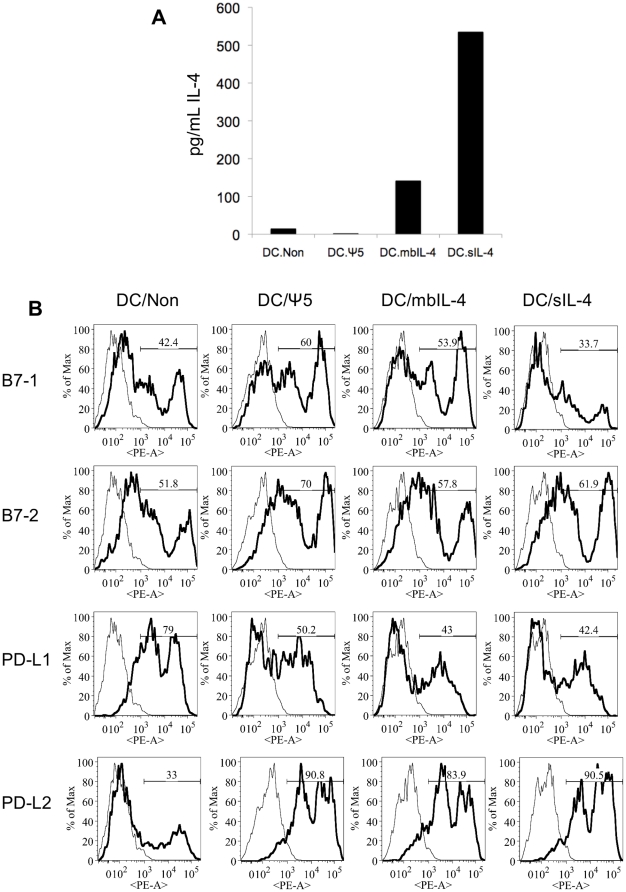
Characterization of DC used for adoptive cellular therapy. DC transduced with adenoviral vectors expressing soluble IL-4 (DC/sIL-4), membrane-bound IL-4 (DC/mbIL-4), or empty adenoviral vector (DC/ψ5) were compared to non-transduced DC (DC/Non) **A**.) After harvest on day 8, 1×10^6^ DC were plated per well in OPTI-MEM media a 96-well tissue-culture plate. Following 24 hours of culture, supernatants were analyzed by ELISA for levels of IL-4 secretion. **B**.) After harvest on day 8, DC were collected and analyzed by flow cytometry. Plots were based on live DC as determined by FSC vs. SSC profiles. Thin lines represent the isotype control stained samples, and bold lines represent staining with anti-B7-1, -B7-2, -PD-L1, or -PD-L2 antibodies, as shown. The percentage of cells in the sample with positive staining is displayed.

We and others have demonstrated previously that bone marrow derived DC genetically modified to express IL-4 reduce disease pathology in mouse models of collagen-induced arthritis and delayed-type hypersensitivity [19], [20]. In arthritis and hypersensitivity models, we found that DC and DC-derived exosomes modified to express either soluble or membrane-bound IL-4 were equally capable of ameliorating disease, indicating that the delivery of high levels of IL-4 alone were not the sole driving factor in the therapeutic effect [20]. To generate DC expressing IL-4, bone marrow derived DC were transduced with an adenoviral vector expressing either soluble (sIL-4) or membrane bound IL-4 (mbIL-4). After transduction, we observed significantly higher levels of IL-4 in the supernatant of DC/sIL-4 than in the control or membrane IL-4 transduced DC culture conditions (Figure 1A).

To determine if transduction with the adenoviral vectors resulted in significant differences in DC maturation, we next examined the phenotype and maturation status of the DC by FACS. The culturing method results in a population of DC that is highly CD11c (>70%) and CD11b (>50%) positive (Table S1), which was then used for adoptive transfer without further purification. We examined CD40 expression in all four populations, and find that ∼40% of DC were positive for CD40 regardless of whether they were non-transduced or transduced with any one of the three vectors examined (Table S1). We also observed that transduction with a control adenoviral vector (DC/ψ5) increases surface expression of B7-1, B7-2, and PD-L2, and decreased the expression of PD-L1 (Figure 1B). DC/mbIL-4 demonstrated a similar pattern of changes in surface costimulatory molecule expression. However, DC/sIL-4 have decreased expression of B7-1 compared to the DC/ψ5 or DC/mbIL-4, with levels similar to those seen in the non-transduced DC. Although transduced DC upregulate costimulatory molecules due to adenoviral infection, the high levels of IL-4 secreted by the DC/sIL-4 appears to affect the balance of B7 molecules expressed by the DC. This is consistent with the previous observation that IL-4 regulates cytotoxic CD8+ T cell responses by modulating the levels of B7-1 and B7-2 on DC [21].

### DC expressing soluble IL-4 delay the onset of hyperglycemia in 12-week-old NOD mice

**Figure 2:**
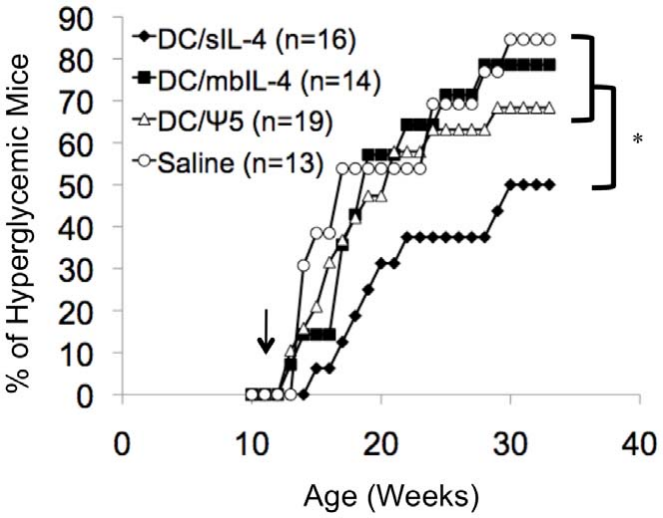
Incidence of diabetes in normoglycemic mice following DC therapy. Mice were screened beginning at 11-weeks of age, and only mice with blood glucose measurements less than 150 mg/dL were randomized to receive a single tail vein injection of either 1×10^6^ DC/sIL-4, 1×10^6^ DC/mbIL-4, 1×10^6^ DC/ψ5, or saline. Combined results from 3 identical, independent experiments are shown. * denotes significance of p≤0.05 by log rank test of DC/sIL-4 compared to saline-treated group. The p-value of comparisons between the DC/mbIL-4, DC/ψ5, and saline-treated groups is greater than 0.05.

Female NOD/ShiLTJ mice were screened beginning at 11 weeks of age for the development of hyperglycemia. Most NOD mice will develop severe insulitis by 10 weeks of age, and, in our experiments, a small percentage (<10%) of mice develop overt hyperglycemia by 12 weeks of age [1]. At 12 weeks, mice with blood glucose measurements <150 mg/dL were randomized to receive a single tail vein injection of 10^6^ DC/sIL-4, 10^6^ DC/mbIL-4, 10^6^ DC/ψ5, or saline alone. Blood glucose levels of mice were followed weekly and the percentage of mice developing hyperglycemia is shown in Figure 2. Treatment with DC/sIL-4 conferred significant protection (p≤0.05 using the log-rank test) with 50% of mice treated with DC/sIL-4 developing diabetes by 30 weeks as compared to >90% of saline-treated mice. There was no therapeutic benefit observed in mice treated with DC/mbIL-4 or DC/ψ5, which were not statistically significant from mice treated with saline alone (p>0.05 for both groups using log-rank test).

### Evaluation of insulitis by histological analysis

**Figure 3:**
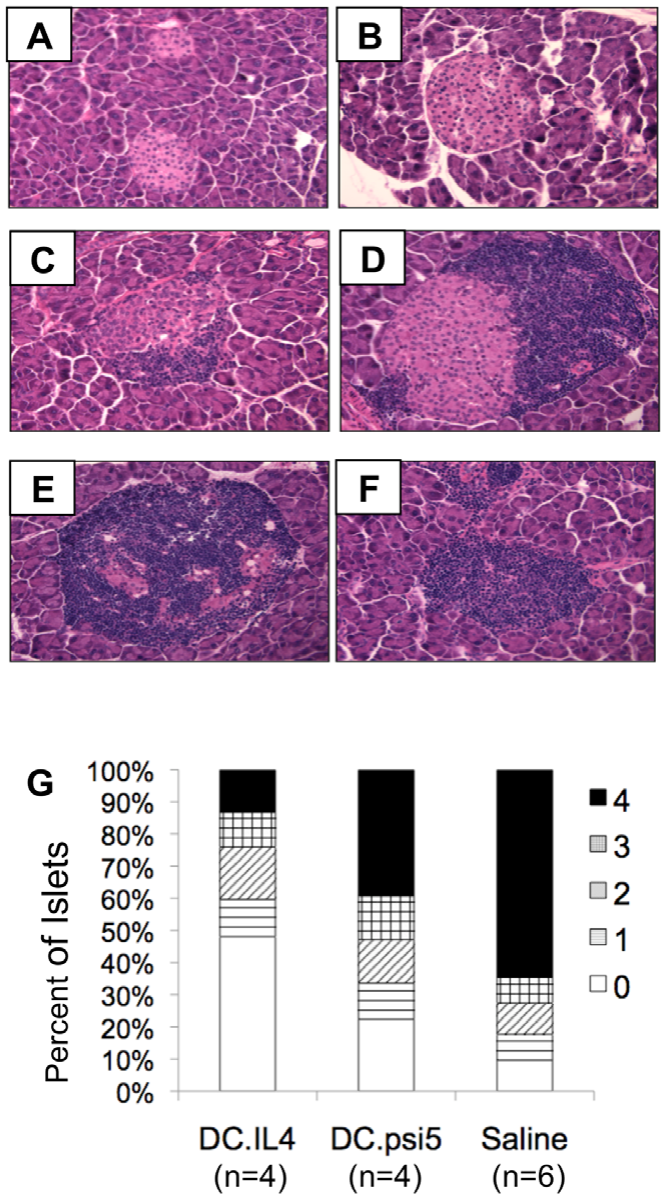
Mononuclear infiltration in mice following adoptive DC gene therapy. Normoglycemic mice received a single tail vein injection of either 1×10^6^ DC/sIL-4, 1×10^6^ DC/ψ5, or saline at 12 weeks of age and were monitored. At 15 weeks of age, mice with normal blood glucose levels were sacrificed. Photomicrographs were taken of hematoxylin and eosin stained sections of pancreata collected from these mice. Mice treated with DC/sIL-4 show little or mild mononuclear infiltration surrounding the islets (**A–B**), whereas DC/ψ5 treated mice have moderate islet infiltration in the majority of islets (**C–D**), and the majority of mice treated with saline have severe insulitis (**E–F**). **G**.) The insulitis score was determined by a blinded investigator using the following scale: 0 = no lymphocytic infiltration; 1 = peri-insultits; 2 = insulitis affecting less than 33% of the islet area; 3 = insulitis affecting 33%–66% of the islet area; 4 = insluitis affecting greater than 66% of the islet area. A minimum of 30 islets were evaluated per mouse on a minimum of 3 slides at least 100 µm apart. Statistical comparison between the groups using Student's t-test demonstrates: DC/sIL4 vs. Saline p = 0.024, DC/sIL/4 vs. DC/ψ5 p = 0.167, DC/ψ5 vs. Saline p = 0.293.

12-week old normoglycemic mice received a single tail vein injection of 10^6^ DC/sIL-4, 10^6^ DC/mbIL-4, 10^6^ DC/ψ5, or saline alone and sacrificed at 15 weeks of age. Insulitis was evaluated by histology in order to determine the effect of treatment with DC/sIL-4 on mononuclear cell infiltration into the islets. Three weeks following treatment, mice treated with DC/sIL-4 had sparsely infiltrated islets, with a majority of islets demonstrating no mononuclear cell infiltration or peri-insulitis (Figure 3A & B). In contrast, mice treated with DC/ψ5 had infiltrative insulitis in slightly greater than half of the islets (Figure 3C & D). Mice receiving saline alone had infiltrative insulitis in greater than 75% of all islets examined, with 50% of all islets completely infiltrated (Figure 3E &F). The insulitis score (Figure 3G) at 15 weeks of age in DC/sIL-4 recipient mice was lower (1.45±0.93) than in mice receiving DC/ψ5 (2.51±0.95) or saline (3.19±0.86) and there was a statistically significant difference between the insulitis scores of the DC/sIL-4 treated and saline treated animals (p = 0.024). The percentage of total intact islets and those with mild peri-insulitis in the DC/sIL-4 treatment group was 60%, which was twice the amount seen in the DC/ψ5 treated group (33.6%) and three times as many as that seen in the saline treated group (17.7%).

### DC/sIL-4 treatment increases IL-4 secretion by islet-antigen specific T cells

**Figure 4:**
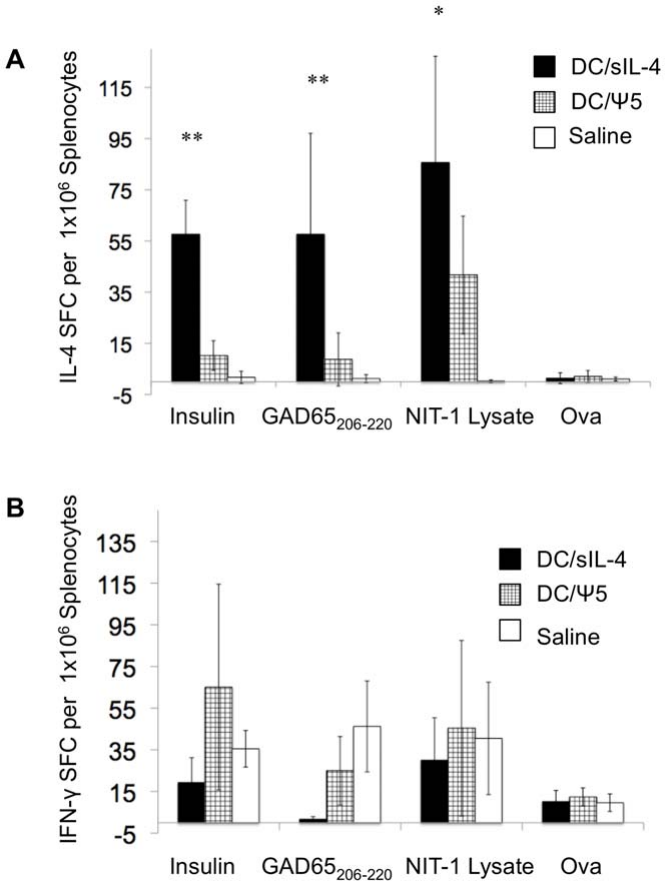
Antigen-specific IL-4 secretion by splenic T cells following treatment with DC overexpressing IL-4. Normoglycemic mice received a single tail vein injection of either 1×10^6^ DC/sIL-4, 1×10^6^ DC/ψ5, or saline at 12 weeks of age and were monitored until 15 weeks of age, when they were sacrificed. Splenocytes were obtained from mice and assessed by ELISPOT for the frequency of splenic T cells secreting IL-4 **(panel A)** or IFN-γ **(panel B)** in response to stimulation with GAD65_206–220_, insulin, and NIT-1 cell lysate. Results shown are representative of four mice per group. Using student's t-test, ** denotes significance at p<0.05 of DC/sIL-4 treated group compared to both DC/ψ5 and saline treated groups, * denotes significance at p<0.05 of DC/sIL-4 group compared to saline alone.

The balance of Th1 and Th2 cytokines is important for the progression of autoimmune diseases, and in type 1 diabetes, a Th1-skewed balance of T cell responses contributed to the pathogenesis and progression of the disease [22], [23], [24]. Treatment with IL-4 has been shown to maintain Th2 CD4+ T cell responses that inhibit the progression of diabetes. Thus we investigated whether DC/sIL-4 treatment had a beneficial effect on Th1/Th2 balance [4]. Normoglycemic 12-week-old NOD mice were treated with 10^6^ DC/sIL-4, 10^6^ DC/mbIL-4, 10^6^ DC/ψ5, or saline alone and at 15 weeks of age the frequency of splenic cells secreting IFN-γ or IL-4 in response was assessed by ELISPOT. Insulin, Gad65_206–222_, and NIT-1 (a NOD-derived β-cell line) cell lystate were used to stimulate islet-specific cytokine secretion in the splenocytes from treated mice. We did not observe significant changes in the numbers of splenocytes secreting the Th1-accociated cytokine IFN-γ between treatment groups (Figure 4B). In contrast, splenocytes from DC/sIL-4 treated mice exhibited a significant increase in the secretion of the Th2-associated cytokine IL-4 in response to all three antigens tested (Figure 4A). These results showing significant increases in the ratio of Th2/Th1 specific T cells after treatment with DC/sIL-4 in all three stimulation conditions demonstrate that administration of DC/sIL-4 is able to induce changes in Th1/Th2 balance within NOD mice.

### DC/sIL-4 treatment upregulates FoxP3 expression

Three and four weeks following treatment with DC/sIL-4, mice were sacrificed and splenocytes analyzed by FACS to determine the proportion of CD4+ CD25+ FoxP3+ regulatory T cells within the spleen. Cells were analyzed for expression of CD4, and results are expressed as the ratio of CD4+ CD25+ FoxP3+ to the total number of CD4+ splenocytes. The results suggest that DC/sIL-4 treatment did not have any effect the levels of FoxP3+ regulatory T cells within the spleen.

**Figure 5:**
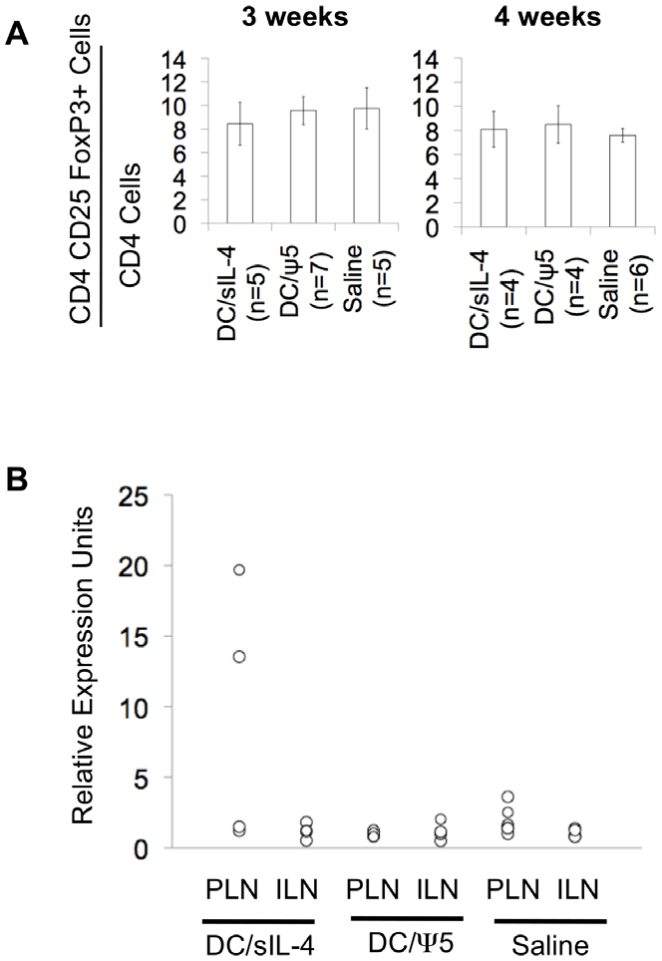
Analysis of regulatory T cell induction by DC/sIL-4 treatment. Normoglycemic mice received a single tail vein injection of either 1×10^6^ DC/sIL-4, 1×10^6^ DC/ψ5, or saline at 12 weeks of age and were monitored until 15 or 16 weeks of age, when they were sacrificed. **A**.) Splenocytes were obtained from mice at 15- or 16-weeks of age and analyzed by FACS for expansion of the CD4+CD25+FoxP3+ regulatory T cell compartment. Splenocytes were stained using isotype control and monoclonal antibodies to CD4, CD25 and FoxP3. Gates were set around live cells using FSC vs. SSC, then around CD4+ cells. Isotype control staining was used to set the FoxP3 gating, and the number of CD4+CD25+FoxP3+ cells per sample were collected and are represented as a ratio over the number of total CD4+ cells counted in each sample. **B**.) FoxP3 gene expression from pancreatic (PLN) and inguinal lymph nodes (ILN) obtained from mice at 15 weeks of age was analyzed using real-time PCR. Mice had been treated with 1×10^6^ DC/sIL-4 (n = 4), 1×10^6^ DC/ψ5 (n = 4), or saline (n = 6) at 12 weeks of age by a single tail vein injection. Expression of FoxP3 was normalized to the expression of beta-actin to give the relative expression, and samples from all mice were then normalized to the mean of the saline-treated ILN group.

We next examined the relative changes in gene expression in the pancreatic lymph nodes of mice three weeks after treatment with DC/sIL-4 or controls. The pancreatic lymph nodes (PLN) play an important role in the priming of inflammation in type 1 diabetes, and it has been demonstrated in numerous studies that adoptively transferred DC are capable of homing to both the spleen and PLN [15], [25]. In a recent study comparing the gene expression profiles of NOD and NOD.B10 mice, it was demonstrated that NOD mice lose PLN expression of IL-4 over time compared to their NOD.B10 counterparts [6]. Thus we were interested in determining if treatment with DC/sIL-4 would result in corresponding changes in the levels of gene expression when compared to DC/ψ5 or saline treated animals. In particular, we examined the levels of IL-4, IFN-γ, and FoxP3 expression in the PLN and inguinal lymph nodes (ILN) of treated mice by quantitative real-time PCR (qRT-PCR). The inguinal lymph nodes were used as a control to ensure that there was not a general effect of DC-based therapy on gene expression in all lymph nodes. Indeed, in all of the experiments, the levels of gene expression in the inguinal lymph nodes of mice were equivalent between groups. Also, there were no changes in levels of IL-4 or IFN-γ expression within the PLN of treated animals at four weeks post-treatment. However, we did detect robust upregulation of FoxP3 in the PLN of half of the DC/sIL-4 treated mice (Figure 5B). This result is consistent with induction of a regulatory T cell phenotype or recruitment of regulatory T cells into the local environment.

### A single administration of DC/sIL-4 reduces onset of hyperglycemia in prediabetic mice

Although many interventions prevent the onset of type 1 diabetes in animal models, few are effective when administered late in the disease process [26]. Therefore, we examined whether DC/sIL-4 therapy was effective at preventing the onset of hyperglycemia when administered in the later stages of disease.

**Figure 6:**
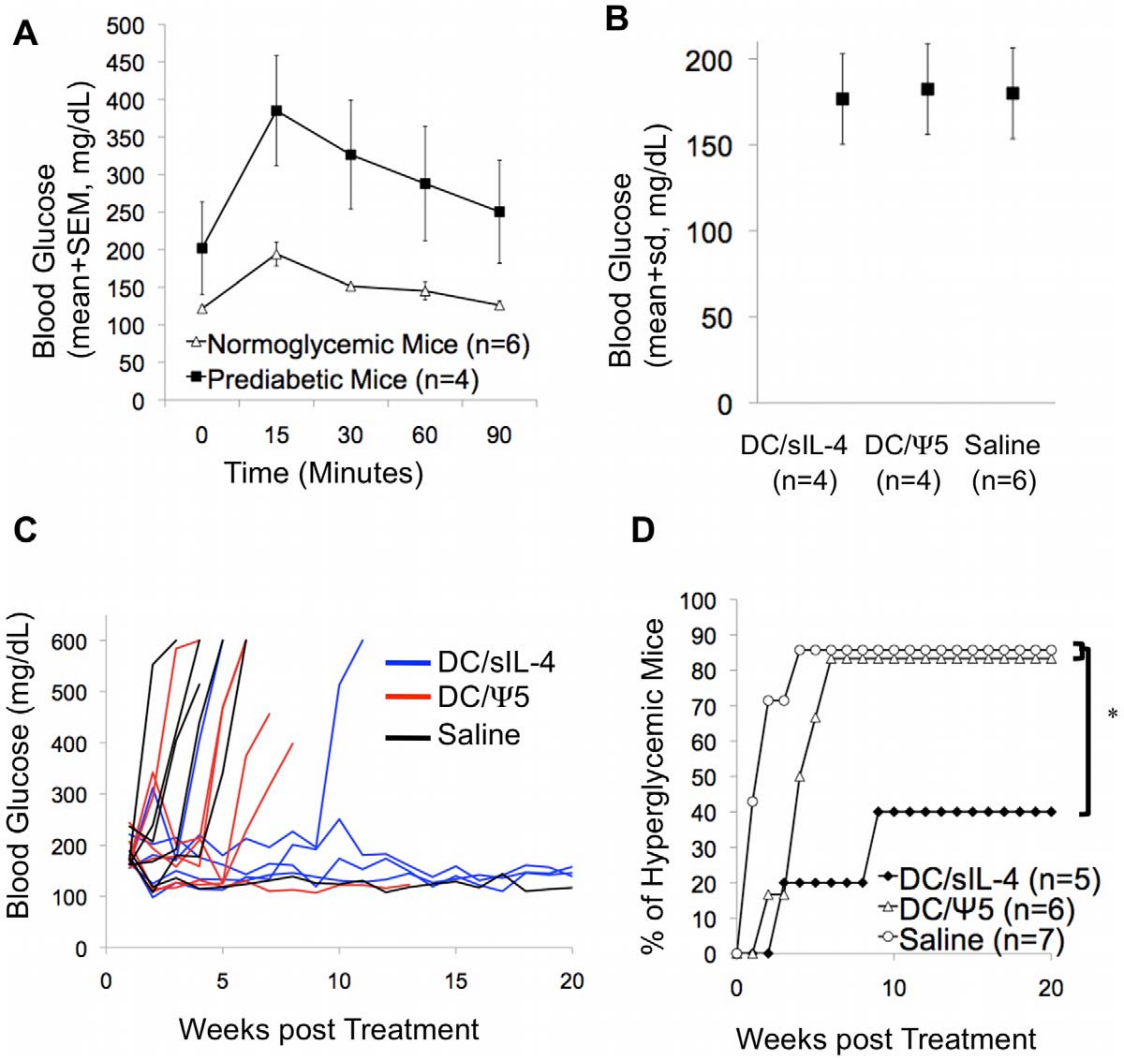
Incidence of diabetes in prediabetic mice following DC therapy. Mice were screened beginning at 11-weeks of age, and mice with blood glucose measurements between 150 and 250 mg/dL were considered prediabetic while mice with blood glucose measurements below 150 mg/dL were considered normoglycemic. **A**.) Normoglycemic and prediabetic mice were injected with 2mg of L-Dextrose per gram of body weight, and their blood glucose measurements were measured at 15, 30, 60, and 90 minutes post injection. **B**.) Mice were randomized into groups in order to avoid treatment bias based on the initial blood glucose. There were no significant differences in the blood glucose of mice prior to receiving treatment. By Student t-test: DC/sIL-4 vs DC/Ψ5 p = 0.703; DC/Ψ5 vs. Saline p = 0.8937; DC/sIL-4 vs Saline p = 0.8183. **C**.) Female prediabetic mice between 12 and 16 weeks of age were randomized to receive a single tail vein injection of either 1×10^6^ DC/sIL-4, 1×10^6^ DC/ψ5, or saline, and were then monitored for the development of hyperglycemia. The results represent the individual responses of each animal treated. **D**.) Kaplein-Meier survival analysis was performed to examine differences in response to treatment by group. * denotes significance of p≤0.05 by log-rank test of DC/sIL-4 compared to DC/ψ5, and saline-treated groups. The p-value of comparisons between the DC/ψ5 and saline-treated groups is greater than 0.05.

12–16 week-old female NOD/ShiLTJ mice were monitored for the development of prediabetes, defined as repeat random blood glucose measurement between 150 mg/dL and 250 mg/dL. Intraperitoneal glucose tolerance testing of prediabetic 14-week-old mice showed an abnormal response to glucose during challenge, with higher peak levels of blood glucose compared to mice in the normoglycemic group (Figure 6A). In addition, at 90 minutes, the blood glucose levels of the prediabetic group had still not returned to baseline, indicating an advanced state of insulitis and islet dysfunction.

The prediabetic mice were randomized to receive a single tail vein injection of 10^6^ DC/sIL-4, 10^6^ DC/ψ5, or saline alone immediately after being classified as prediabetic. As shown in Figure 6B, mice were randomized between groups such that there were no significant differences in the starting blood glucose between the groups. Mice were monitored weekly for the onset of hyperglycemia, and mice treated with DC/sIL-4 maintained euglycemia or demonstrated a delay in the onset of hyperglycemia (Figure 6C). As shown in Figure 6D, DC/sIL-4 treatment significantly reduced the number of mice developing hyperglycemia compared to mice receiving DC/ψ5 or saline (p≤0.05 by Kaplan-Meier analysis). This result clearly indicates that even in more advanced stages of disease, DC/sIL-4 treatment can be an effective therapy to reduce the onset of type 1 diabetes.

## Discussion

A large number of interventions are effective at preventing the onset of diabetes in young NOD mice. However, very few are effective at preventing the onset of hyperglycemia when administered after 10 weeks of age or when administered once disease onset has occurred [18], [26]. Similar to previous results, we demonstrate the ability of IL-4 overexpressing DC to prevent diabetes onset in the NOD model [6], [7]. Our data indicate that DC/sIL-4 are capable of delaying disease onset (Figure 2) and reducing mononuclear infiltrate (Figure 3) in normoglycemic, but insulitic 12-week old mice. Our results are consistent with a recent report demonstrating that transduction of DC with a lentiviral vector expressing IL-4 prevents the onset of diabetes in 12-week old NOD mice [6]. We have extended these initial observations, now demonstrating that IL-4 transduced DC can prevent the onset of hyperglycemia in a significant number of prediabetic mice (Figure 6B). Screening NOD mice for elevated blood glucose allowed us to efficiently select mice with impaired response to glucose challenge, which is indicative of the most advanced stages of insulitis (Figure 6A) [27]. Interestingly, our results indicate that DC/sIL-4 may be more effective at reversing disease onset in the prediabetic NOD mice (60% diabetes free at 32 weeks of age compared to 14% of saline-treated mice) than 12-week-old normoglycemic mice (50% diabetes free at 32 weeks of age compared to 14% of saline-treated mice). In addition, our results suggest that at least part of the therapeutic effect of the DC/sIL-4 is mediated through the induction of FoxP3-positive regulatory T cells.

The therapeutic effect of the DC/sIL-4 in prediabetic NOD mice was dependent upon their ability to secrete soluble IL-4, as we do not see any therapeutic benefit in mice receiving the DC/mbIL-4. This is in contrast to our previous results in arthritis and hypersensitivity models, where DC/mbIL-4 are equally effective at reducing inflammation as DC/sIL-4 [20]. The difference between these model systems and the NOD model of type 1 diabetes perhaps indicates that the role of the therapeutic DC in this model is to deliver high levels of IL-4 to critical areas of disease activity, such as the PLN. As shown by Creusot et al., NOD mice have a significant reduction of IL-4 levels in their PLN compared to NOD.B10 controls, and DC transduced to express IL-4 effectively home to the PLN and correct this deficiency, at least in the short-term [6]. Our data are consistent with the finding that high levels of IL-4 secretion are required by the transduced DC in order for a delay in diabetes onset to be seen.

Use of replication-deficient adenoviral gene vectors to deliver IL-4 to DC resulted in increased IL-4 expression by DC as well as an altered DC costimulatory molecule phenotype (Figure 1). Our data indicate that adenoviral infection results in upregulation of the costimulatory molecules B7-1, B7-2, and PD-L2, consistent with other studies evaluating adenoviral gene transfer to DC [28], [29]. We further observed that the high levels of IL-4 secreted into the culture media result in nearly identical expression levels of B7-1 on the DC/sIL-4 and non-transduced DC. This is consistent with a previous report indicating that IL-4 treatment of DC leads to upregulation of B7-2 and downregulation of B7-1, and that DC treated with recombinant IL-4 decrease in the activity of diabetogenic CD8+ cells in a manner dependent on B7-2 on the DC [21]. Although viral transduction induces changes in expression of DC costimulatory molecules, we hypothesize that overexpression of IL-4 mitigates some of these effects, and the resulting increased B7-2∶B7-1 ratio may contribute to the overall therapeutic effect of the DC/sIL-4 *in vivo*. Further previous studies confirm that MHC Class I and II deficient DC transduced to express soluble IL-4 do not delay diabetes onset, confirming that there is indeed a critical role for antigen presentation by the transduced DC for therapeutic effects to be seen [6].

Our analysis of the mechanism of the therapeutic effect of DC/sIL-4 demonstrated that DC/sIL-4 administration to NOD mice results in increased islet-antigen specific secretion of the Th2-cytokine IL-4, whereas there were no changes in the levels of secreted Th1 cytokine IFN-γ (Figure 4). We hypothesize that this represents an alteration of the T cell responses within the treated animal, and does not represent persistence of the DC/sIL-4 *in vivo* for several reasons. First, ELISPOT assays were conducted three weeks following the administration of the DC/sIL-4. Previous studies using DC expressing firefly luciferase as a reporter gene indicate that DC selectively traffic to the spleen and pancreatic lymph node of NOD mice and expression of luciferase in these tissues suggests the numbers of DC peak in these tissues between day 1 and day 3 and then wane significantly by day 8 post-injection [6]. Secondly, the frequency of the observed IL-4 spot forming units in our assays significantly exceeds the original dose of 10^6^ DC/sIL-4 administered to the mice.

qRT-PCR analysis revealed that FoxP3 was significantly upregulated in the PLN of half of the mice receiving DC/sIL-4 (Figure 5B). IL-4 has been shown to play a role in the extrathymic induction of FoxP3+ regulatory T cells [30]. In addition, we have previously shown that transduction of the islets with an AAV vector expressing IL-4 under the control of the mouse insulin promoter results in expansion of the percentage of CD4+CD25+FoxP3+ regulatory T cells as opposed to saline-treated control animals [5]. However, splenocytes of DC/sIL-4 treated mice showed no significant changes in the percentages of CD4+CD25+FoxP3+ T-cells when compared to control treated animals either three or four weeks following treatment (Figure 5A). Thus the induction of regulatory T cells in the PLN may predict which animals respond positively to DC/sIL-4 therapy.

We previously have demonstrated that exosomes secreted by DC transduced with IL-4 are therapeutic in mouse models of delayed-type hypersensitivity and arthritis [20]. Despite a growing number of studies of exosome-based therapy for autoimmune disease, there have been no studies exploring the therapeutic potential of exosomes for type 1 diabetes to date. However, in contrast to our previous results, in which a dose of 1 µg was effective at reducing disease when administered systemically by tail vein in collagen-induced arthritis and experimental autoimmune encephalomyelitis models [20], 1 µg of DC-derived exosomes was not capable of delaying the onset of diabetes in NOD mice (data not shown).

Collectively, our data demonstrate that adoptive transfer of DC/sIL-4 not only reduces the onset of diabetes in 12-week-old NOD, but can also maintain euglycemia in prediabetic mice ages 12–16 weeks of age that have demonstrated abnormalities in glucose homeostasis. This is the first demonstration that DC/sIL-4 are capable of preventing disease onset at such a late stage of insulitis, and provides further confirmation that this approach is clinically relevant. A patient's own DC could be harvested and modified to express IL-4 *ex vivo*, then administered via intravenous injection. As identification of patients at risk for type 1 diabetes improves, it may become increasingly possible to use a cell-based therapy such as DC/sIL-4 to prevent onset of disease in at risk individuals, or perhaps to intervene at the time of clinical diagnosis.

## Materials and Methods

### Mice

Female NOD/ShiLTJ mice were purchased from Jackson Laboratory (Bar Harbor, ME) and maintained in a specific pathogen-free facility at the University of Pittsburgh according to US Department of Agriculture and National Institutes of Health guidelines. All experiments were conducted under protocols reviewed and approved by the Institutional Animal Care and Use Committee.

### Vector construction and Adenovirus Generation

Adenoviruses expressing soluble murine IL-4 (Ad.sIL-4) and membrane-bound (Ad.mbIL-4) and were constructed, propagated, and titered according to standard protocols as previously described [31]. The membrane-bound IL-4 construct is composed of IL-4 fused to the transmembrane domain of CD80. Recombinant adenoviruses were generated by homologous recombination in 293 cells expressing Cre recombinase (CRE8 cells), after co-transfection with a plasmid (ψ5) expressing the adenovirus 5-derived, E1- and E3-deleted adenoviral backbone and pAdlox, the adenoviral shuttle vector that expresses either sIL-4 or mbIL-4. Recombinant adenoviruses were purified by CsCl gradient ultracentrifugation, dialyzed in sterile virus storage buffer, aliquoted and stored at −80°C.

### DC Generation

BMDC were prepared following a bulk-culture protocol modified as previously described [32], [33]. Briefly, bone marrow was collected from tibias and femurs of 6- to 7-week-old female NOD/ShiLTJ mice. Contaminating erythrocytes were lysed, and monocytes were collected from the interface after centrifuging on Nycoprep (NycoMed, Roskilde, Denmark) at 600×g for 20 min at RT. Cells were cultured for 24 h in complete media (RPMI 1640 containing 10% FBS, 50 µM 2-β-Mercaptoethanol, 2 mM glutamine, 0.1 mM nonessential amino acids, 100 IU/mL penicillin/streptomycin) to remove adherent macrophages. Non-adherent cells were placed in fresh growth media (complete media containing 1000 U/mL of GM-CSF and IL-4) to generate DC. Cells were cultured for 4 days then harvested for adenoviral (Ad) transduction. For Ad infection, 1×10^6^ DC were incubated with 5×10^7^ PFU of the virus in 1mL of serum-free media for 2 hours, and then 10 mL complete media was added to the cells. After overnight incubation, DC were washed vigorously three times in PBS to remove excess virus and cultured for an additional 48 h in growth media. On day 8, DC were recovered and used for adoptive transfer or in vitro experiments. This infection method routinely gives us ∼70–80% transfection efficiency using Ad.eGFP as a control [34].

### FACS Analysis of DC

Dendritic cells were preincubated with anti-CD16/32, then stained with 1–2 µL of PE-labeled mAbs (B7-1, B7-2, PD-L1, PD-L2, CD40, CD11b, and CD11c, all from eBioscience, San Diego, CA) in 100 µL total volume of ice-cold PBS containing goat serum. Following staining, cells were washed twice in FACS buffer, resuspended in 400 µL FACS buffer, and examined by FACS. Results were analyzed using FlowJo (Treestar Inc. Ashland, OR).

### ELISA Analysis of DC Supernatant

Supernatants from DC were collected and analyzed using a sandwich ELISA to detect IL-4 (eBioscience San Diego, CA) according to the manufacturer's protocol. The limits of detection were 4 pg/mL.

### Adoptive Transfer of DC to NOD/ShiLTJ mice

Blood glucose levels of NOD/ShiLTJ mice were monitored using blood drawn from the tail vein and analyzed via a glucometer (Ascencia BREEZE; Bayer). Mice with blood glucose readings below 150 mg/dL were considered normoglycemic. Mice with repeat measures between 150 and 250 mg/dL on consecutive days were considered prediabetic, and mice with repeat measures greater than 300 mg/dL on consecutive days were considered hyperglycemic.

To test the ability of treatment to delay diabetes onset, normoglycemic 12-week old female NOD/ShiLTJ mice were randomized to receive a single tail vein injection with either 10^6^ DC or PBS. For studies examining the ability of DC therapy to prevent diabetes in prediabetic mice, mice 12–16 weeks of age with repeat blood glucose measurements between 150 and 250 mg/dL were randomized to receive a single tail vein injection containing either 10^6^ DC therapy or PBS alone.

### Intraperitoneal Glucose Tolerance Test

Mice were fasted for 12 hours, and then weighed. Baseline blood glucose measurement was determined prior to i.p. injection with L-Dextrose suspended in PBS at a dose of 2 mg per gram of body weight. Repeat blood glucose measurements were determined at 15, 30, 60, and 90 minutes following dextrose injection.

### Histology and Islet Grading

Pancreata were collected from 15-week old mice following DC therapy at 12 weeks of age, and were fixed, and embedded. Only mice that had remained normoglycemic by 15 weeks of age were used for histology studies. 5 µm sections were deparaffinized, stained with hematoxylin and eosin, and evaluated by a blinded investigator for infiltrating mononuclear cells within the islets. Individual islets were assigned scores based on their appearance: 0 = no lymphocytic infiltration; 1 = peri-insultits; 2 = insulitis affecting less than 33% of the islet area; 3 = insulitis affecting 33%–66% of the islet area; 4 = insulitis affecting greater than 66% of the islet area. The histology score index per mouse was calculated by dividing the sum of all individual islets scored by the total number of islets evaluated, and was used to represent each mouse in statistical testing. A minimum of 30 islets were evaluated per mouse on a minimum of 3 slides at least 100 µm apart.

### Evaluation of splenic antigen-specific responses by ELISPOT and splenic regulatory T cell levels

15-week old mice were sacrificed following DC therapy at 12 weeks of age. Spleens were collected and splenocytes immediately extracted. Contaminating erythrocytes were lysed with lysis buffer.

For ELISPOT assay, 5×10^5^ splenocytes per well were stimulated with GAD65_206–220_ (50 ug/mL, Anaspec, Freemont, CA), insulin (50 ug/mL Sigma-Aldrich, St. Loius, MO), NIT-1 freeze-thaw cell lysate (20 ug/mL), or ovalbumin (OVA) protein (50 ug/mL Sigma-Aldrich, St. Loius, MO) in serum-free media in 96-well ELISPOT plates precoated with an anti-IL-4 or anti-IFN-γ mAbs. ELISPOT plates were cultured for 36 hours followed by incubation with biotin conjugated-anti-IL4 or anti-IFN-γ mAb, and development with streptavidin-peroxidase and 3-amino-9-ethylcarbazole according to manufacturer's recommendations (BD Biosciences, San Jose, CA). Spots were counted with an ImmunoSpot counter (Cellular Technology, Shaker Heights, OH). Stimulations were conducted in triplicate per mouse per antigen.

To evaluate levels of splenic T regulatory cells, splenocytes were surface stained with antibodies for CD4 and CD25 for 30 minutes at 4°C according to the manufacturer's recommendations (eBioscience, San Diego, CA). Cells were washed then incubated in the dark at 4°C in fixation/permeabilization buffer overnight. Cells were again washed and resuspended in permeabilization buffer, and incubated in the dark at 4°C for 1 hr with an anti-FoxP3 monoclonal antibody (clone FJK-16s, eBioscience). Cells were washed twice with permeabilization buffer, then resuspended with FACS buffer and examined by FACS.

### Quantitative PCR

15-week old mice were sacrificed following DC therapy at 12 weeks of age. Pancreatic lymph nodes (PLN) were harvested and RNA isolated according to manufacturer's recommendations using an RNAqueous-Micro Isolation Kit (Ambion, Austin TX). 375 ng RNA was used as a template to generate cDNA using SuperScript III First-Strand Synthesis Kit for qRT-PCR (Invitrogen, Carlsbad, CA). Real-time PCR was done using SYBR GreenER qPCR SuperMix (Invitrogen) kit on an iQ5 device (Biorad, Hercules, CA). The SuperMix kit uses Fluorescein to normalize the fluorescence fluctuations between reactions. Primers used for qRT-PCR were: GACGGCCAGGTCATCACTAT (ß-actin forward), AAGGAAGGCTGGAAAAGAGC (ß-actin reverse), TCTTGCCAAGCTGGAAGACT (FoxP3 forward), GGGGTTCAAGGAAGAAGAGG (FoxP3 reverse).

### Statistics

Survival analysis was conducted using Kaplan-Meier analysis and a log-rank test for significance between groups on Stata statistical software (College Station, TX). Other results were compared by Student's t test or ANOVA with Fisher's Least Significant Difference post-hoc test using SPSS statistical software (Chicago, IL). P values≤0.05 were considered statistically significant.

## Supporting Information

Table S1 Characteristics of Prediabetic Mouse Experiments. DC transduced with adenoviral vectors expressing soluble IL-4 (DC/sIL-4), membrane-bound IL-4 (DC/mbIL-4) or empty adenoviral vector (DC/Ψ5) were compared to non-transduced DC (DC/Non). After harvest on day 8, DC were collected and analyzed by flow cytometry. Gates were set on live DC as determined by FSC vs. SSC profiles. The percentage of cells in each sample that was positive for the respective marker above the baseline isotype control staining is shown in the table. These results are representative of a single DC preparation.
(0.13 MB TIF)
