# Supplementary figures and images for: Dendritic Cells Transduced to Express Interleukin 4 Reduce Diabetes Onset in Both Normoglycemic and Prediabetic Nonobese Diabetic Mice
Source: PLoS One. 2010 Jul 29;5(7):e11848. doi: 10.1371/journal.pone.0011848 (PMC2912295; doi:10.1371/journal.pone.0011848)

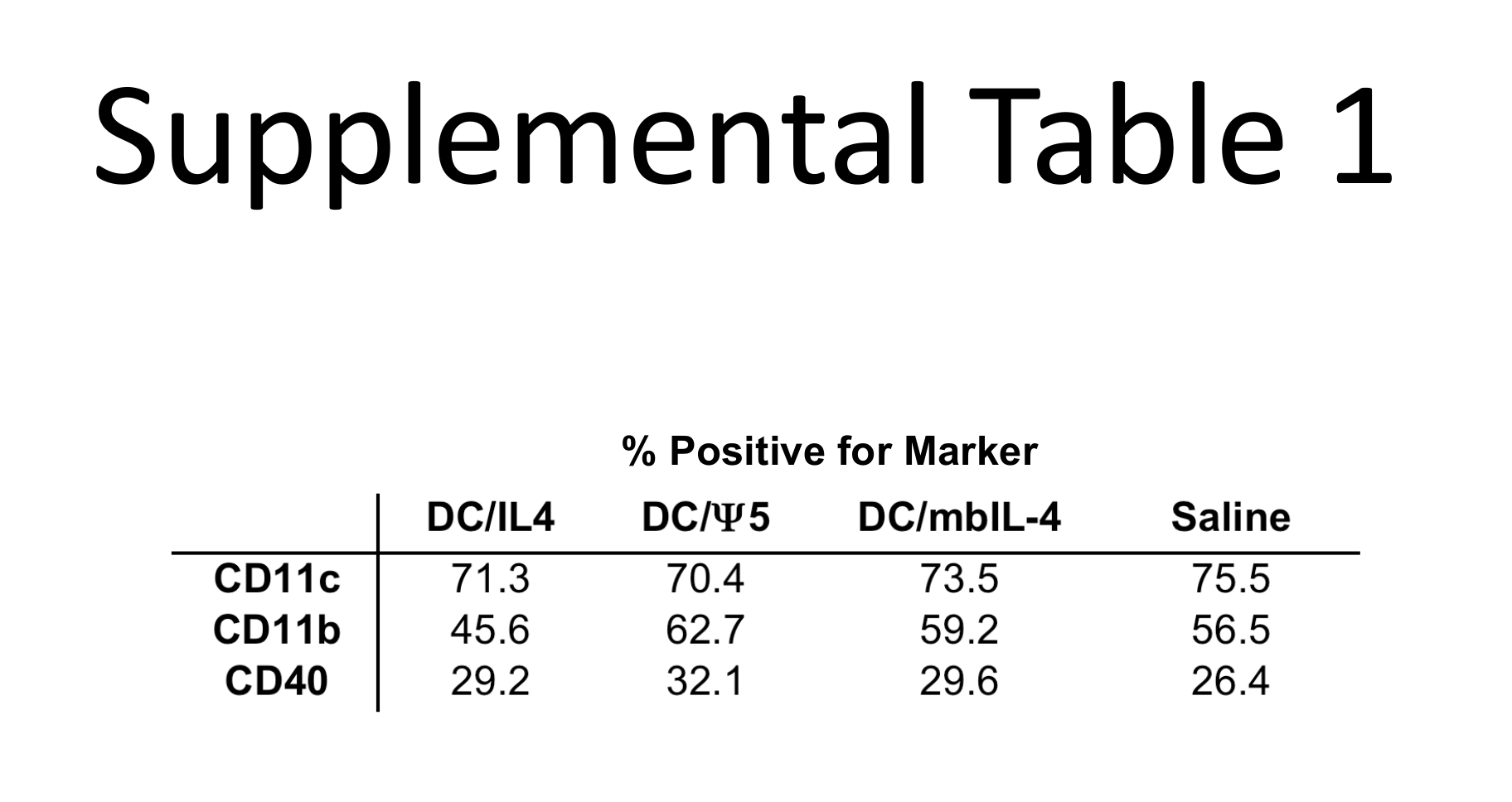

Supplement: Table S1 — Characteristics of Prediabetic Mouse Experiments. DC transduced with adenoviral vectors expressing soluble IL-4 (DC/sIL-4), membrane-bound IL-4 (DC/mbIL-4) or empty adenoviral vector (DC/Ψ5) were compared to non-transduced DC (DC/Non). After harvest on day 8, DC were collected and analyzed by flow cytometry. Gates were set on live DC as determined by FSC vs. SSC profiles. The percentage of cells in each sample that was positive for the respective marker above the baseline isotype control staining is shown in the table. These results are representative of a single DC preparation. (0.13 MB TIF) [file pone.0011848.s001.tif]
